# New Insights and Implications of Cell–Cell Interactions in Developmental Biology

**DOI:** 10.3390/ijms26093997

**Published:** 2025-04-23

**Authors:** Guanhao Wu, Yuchao Liang, Qilemuge Xi, Yongchun Zuo

**Affiliations:** State Key Laboratory of Reproductive Regulation and Breeding of Grassland Livestock, Institutes of Biomedical Sciences, School of Life Sciences, Inner Mongolia University, Hohhot 010021, China; 32308026@mail.imu.edu.cn (G.W.); 22308013@mail.imu.edu.cn (Y.L.); qlmgxi@imu.edu.cn (Q.X.)

**Keywords:** cell–cell interactions, embryo, mother-to-fetus binding interface

## Abstract

The dynamic and meticulously regulated networks established the foundation for embryonic development, where the intercellular interactions and signal transduction assumed a pivotal role. In recent years, high-throughput technologies such as single-cell RNA sequencing (scRNA-seq) and spatial transcriptomics (ST) have advanced dramatically, empowering the systematic dissection of cell-to-cell regulatory networks. The emergence of comprehensive databases and analytical frameworks has further provided unprecedented insights into embryonic development and cell–cell interactions (CCIs). This paper reviewed the exponential increased CCIs works related to developmental biology from 2008 to 2023, comprehensively collected and categorized 93 analytical tools and 39 databases, and demonstrated its practical utility through illustrative case studies. In parallel, the article critically scrutinized the persistent challenges within this field, such as the intricacies of spatial localization and transmembrane state validation at single-cell resolution, and underscored the interpretative limitations inherent in current analytical frameworks. The development of CCIs’ analysis tools with harmonizing multi-omics data and the construction of cross-species dynamically updated CCIs databases will be the main direction of future research. Future investigations into CCIs are poised to expeditiously drive the application and clinical translation within developmental biology, unlocking novel dimensions for exploration and progress.

## 1. Introduction

**Figure 1:**
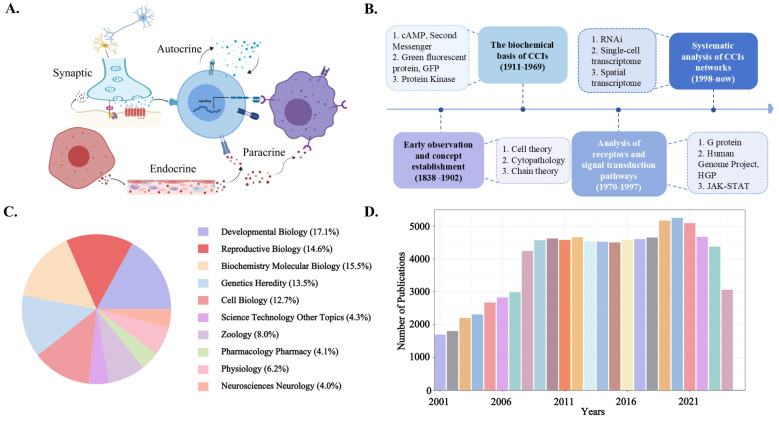
**CCIs types and statistics of recent studies.** (**A**) Schematic of the main modes and mechanisms of CCIs. (**B**) Historic events in CCIs research. A retrospective summary of key events in the development of CCIs up to the present day. This review provides information on historical events. (**C**) Distribution of published papers in different field categories of CCIs in developmental research, 2001–2025 (the data are percentages). (**D**) Statistics on the number of annual published papers in the field of developmental research in CCIs, 2001–2025. Colors are only used to distinguish bars to assist in chart analysis and have no other meanings.

Cell–cell interactions (CCIs) is an intricate progress by which a ligand secreted by a cell (including proteins, metabolites, and small molecule compounds) binds to its own, adjacent, or distant cell surface receptors, which will activate a series of biochemical processes in the target cell, causing it to exhibit a specific biological trait or response [1,2,3]. CCIs assumed a pivotal role during embryonic development by constructing the transmission of signal flows through diverse mechanisms. In accordance with the origin and range of signaling molecules, four recognized CCIs modes have been proposed: endocrine (hormones and other signaling molecules are transported to distant target cells via the circulatory system), paracrine (signaling molecules diffuse to nearby target cells), synaptic transmission (neurotransmitters are released into the synaptic cleft to act on subsequent neurons or effectors), and autocrine (secreted signaling molecules act on the cells that secrete them or cells of the same type). Moreover, these CCIs modes can be further delineated into chemical signal transduction, cell membrane surface contact, and CCIs gap junctions, which correspond to direct and indirect signal transduction pathways, respectively (Figure 1A). During the embryonic development of multicellular organisms, these four modes orchestrate the flow of signals through intricate and dynamic networks, constituting a defining feature of multicellular life forms [4].

Although modern biology has extensively elucidated the diverse modalities of CCIs and their pivotal roles in multicellular organisms, these insights were not achieved overnight. Over nearly two centuries of rigorous scientific inquiry, researchers have continuously refined our understanding of CCIs through a series of groundbreaking discoveries. In the 19th century, Matthias Jakob Schleiden and Theodor Schwann’s cell theory established the fundamental principle that cells constitute the basic structural and functional units of life [5]. Shortly thereafter, Rudolf L.K. Virchow advanced the field with cellular pathology, emphasizing that diseases originate from pathological alterations at the cellular level [6]. Paul Ehrlich’s side-chain theory further unveiled the molecular mechanisms by which cells recognize external signals, laying the conceptual foundation for developmental and molecular biology [7]. With the advent of the 20th century, the rapid evolution of biochemistry and molecular biology facilitated the discovery of key molecules governing cellular functions. Earl Wilbur Sutherland Jr. identified cyclic adenosine monophosphate (cAMP) as a second messenger, elucidating a fundamental mechanism of CCIs signal transduction [8]. Osamu Shimomura’s discovery of green fluorescent protein (GFP) revolutionized molecular biology by enabling real-time visualization of CCIs’ dynamics [9]. Simultaneously, Edwin G. Krebs and Edmond H. Fischer characterized protein kinases, revealing the central role of phosphorylation in CCIs’ signaling networks [10]. This was further expanded by Martin Rodbell and Alfred G. Gilman, who uncovered the G protein-mediated signaling pathway, significantly advancing our comprehension of cellular communication mechanisms [11]. Entering the 1990s, a new wave of discoveries further refined the landscape of CCIs’ research. James E. Darnell, George Stark, and Ian Kerr delineated the JAK-STAT signaling pathway, demonstrating how cytokine-mediated signal transduction directly regulates gene expression [12]. Concurrently, the Human Genome Project (HGP) provided an unprecedented genomic perspective on CCIs, paving the way for advances in systems biology and precision medicine [13]. In the 21st century, technological breakthroughs have propelled CCI research into new dimensions of precision and complexity. Andrew Fire and Craig Mello introduced RNA interference (RNAi), a transformative mechanism for gene silencing and regulation, which opened novel avenues for genetic and therapeutic research [14]. Mark Krasnow and Stephen Quake pioneered single-cell transcriptomics, enabling high-resolution analysis of gene expression at the individual cell level [15]. In parallel, Patrik Ståhl and Joakim Lundeberg spearheaded the development of spatial transcriptomics, which integrates spatial context into transcriptomic profiling, offering unprecedented insights into tissue architecture and cellular microenvironments [16] (Figure 1B).

Collectively, these landmark discoveries have not only shaped our current understanding of CCIs but also provided a robust foundation for advancements in biomedicine, immunology, neuroscience, and developmental biology. As research continues to push the boundaries of CCIs, these foundational discoveries serve as cornerstones for future scientific innovation.

In the past 25 years, these studies cover the fields of cell biology, molecular biology, biochemistry, genetics, and developmental biology, highlighting the broad interdisciplinary potential of CCIs (Figure 1C). CCIs’ research in the context of developmental biology has attracted more attention, and the number of relevant publications has increased year by year, especially in the past ten years (Figure 1D). Collectively, investigation and research show that CCIs provide core insights into developmental biological regulatory mechanisms driven by data and drives future research in this field.

This paper systematically collates 39 databases and 93 analysis tools related to CCIs between 2019 and 2025, trying to interpret the development process and future research direction of CCIs in the field of developmental biology in recent years. We list the major data types and algorithmic frameworks used in CCI studies, including methods based on bulk RNA sequencing (bulk RNA-seq), single-cell RNA sequencing (scRNA-seq), and spatial transcriptome (ST) sequencing data. The key steps of these algorithms (such as data processing, interaction network definition, modeling, and validation) are comprehensively compared below. Finally, this article meticulously explores the complex applications and enduring challenges of CCIs within the realm of developmental biology and presented a forward-thinking outlook on the evolution of data analysis methodologies, along with the development of next-generation databases and analytical tools. Future investigations into CCIs are poised to expeditiously drive the application and clinical translation within developmental biology, unlocking novel dimensions for exploration and progress.

## 2. Research Status of CCIs

### 2.1. Application of High-Throughput Sequencing Technology

CCIs are essential in developmental biology, key for cell differentiation, proliferation, organogenesis, and functional maturation. From 2008, bulk RNA-seq dominated for quantifying tissue or cell-population RNA expression. However, its failure to distinguish individual cell-type transcriptional profiles limited CCIs research. ScRNA-seq has transformed the field. By providing single-cell resolution gene expression data, it reveals cellular heterogeneity and interaction complexity, offering new insights into how CCIs impacts developmental biology [17,18].

By way of illustration, Vento-Tormo et al. [19] utilized scRNA-seq to analyze 70,000 placental and maternal cells during early pregnancy, uncovering cellular characteristics and immune interaction networks at the placental–decidual interface, providing important insights into how CCIs during embryonic development maintains pregnancy. Similarly, Li et al. [20] employed scRNA-seq to investigate the communication networks between distinct germ layers during embryogenesis, elucidating how specific cytokine–receptor interactions guide cell fate determination and organ differentiation in the early stages of development. Building on prior research, another study demonstrated how signal transduction between diverse cell types during Drosophila wing development regulates cell proliferation and differentiation via the JAK/STAT and Notch pathways, ultimately resulting in the formation of functionally specialized wing structures [21]. In plant development research, Shahan et al. [22] applied scRNA-seq to analyze CCIs in Arabidopsis root tip meristematic tissues and discovered how cell type specific communication patterns regulate root growth and differentiation processes at different developmental stages.

Researchers can use scRNA-seq data to identify embryonic tissue’s cellular composition and interactions, revealing cell type roles in development [20,23]. Also, the same cell type shows different gene expression profiles across tissues, highlighting the tissue microenvironment’s impact on cellular states [24]. However, scRNA-seq lacks spatial info, making it hard to track cell origins and interpret CCIs. To address this, ST emerged, allowing accurate cell positioning. Yuan et al. used ST on mouse somatosensory and visual cortices, finding complex interactions between cell types crucial for signal regulation, emphasizing CCI’s role in tissue function [25].

Spatially resolved transcriptomics is groundbreaking for studying biological processes like mammalian embryogenesis. But current ST limitations in resolution, gene capture, and field of view restrict its use on large, three-dimensional late pregnancy embryos [26]. To overcome this, researchers developed Stereo-seq, combining DNA nanosphere arrays with in situ RNA capture for enhanced spatial omics resolution. Stereo-seq created the mouse organogenesis spatiotemporal transcriptome atlas (MOSTA), offering single cell resolution and high sensitivity for mapping mouse organogenesis transcriptional changes. This comprehensive atlas can greatly advance our understanding of normal and pathological mammalian development affected by CCIs [27].

### 2.2. Databases and Tools Related to CCIs

#### 2.2.1. Database Related to CCIs

With the continuous refinement of databases encompassing protein–protein and ligand–receptor interactions, a robust foundation of prior knowledge has been established for advancing CCIs analysis. These databases primarily include data on both simple and complex ligand–receptor interactions, receptor–transcription factor relationships, and other relevant interactions across human and mouse species. Additionally, specialized repositories, such as MACC [28], curate metabolic information pertinent to CCIs. The advent of these prior knowledge databases significantly enhances the accuracy and interpretability of CCIs analyses. Tools such as CellTalkDB [29], CellChatDB [30], and CellPhoneDB [31] exemplify how these resources facilitate the systematic investigation of CCIs. By integrating prior knowledge with multidimensional data—such as bulk RNA-seq, scRNA-seq, and ST—researchers can unravel intricate CCIs’ networks, uncovering pivotal regulatory mechanisms that govern embryonic development. The review provides a comprehensive conclusion of commonly utilized databases for CCIs analysis (Table 1), offering a valuable resource for methodological guidance. Supplementary Table S1 further expands on this by presenting an extensive array of resources to support future study in related fields.

#### 2.2.2. CCIs’ Tools and Analysis Process

**Figure 2:**
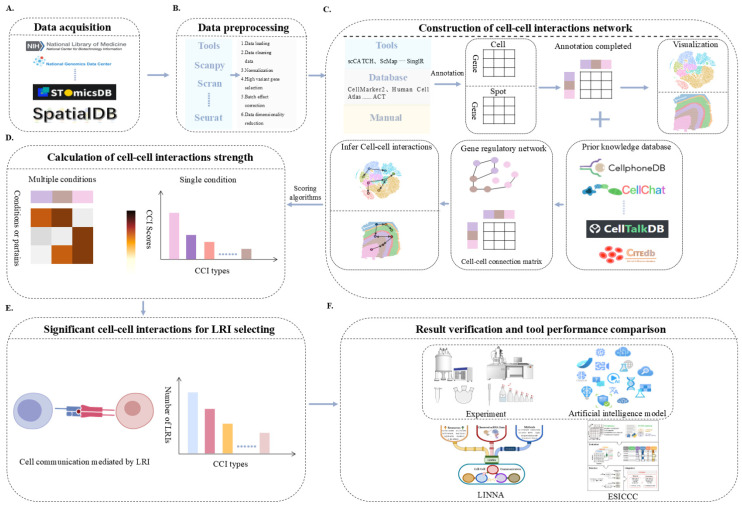
**CCI analysis process.** (**A**) Download the required scRNA-seq or ST data from public databases. (**B**) Preprocess the downloaded data that have been completed. (**C**) Apply tools, databases, or custom methods to annotate cell types on the obtained data expression matrix. Afterwards, existing or self-built prior knowledge bases can be utilized to construct CCIs’ networks. At the same time, some tools do not require prior knowledge base and use deep learning methods to infer CCIs’ networks. (**D**) Calculate CCI strength and select the CCIs with the highest strength. (**E**) Significant CCIs for LRI selecting. (**F**) Result verification and tool performance comparison, divided into experimental verification, artificial intelligence verification, and comparison of tools and methods.

The exponential proliferation of high-throughput sequencing data, coupled with the cumulative experimental accretion of prior knowledge regarding ligand–receptor interactions, has propelled the in-depth decipherment of CCI mechanisms in a remarkable fashion. However, a decisive challenge remains: how to effectively integrate sequencing data with ligand–receptor interactions’ information to systematically elucidate CCIs’ networks and dynamic regulatory mechanisms in developmental biology. This paper meticulously collates and comprehensively summarizes the extant CCI analysis tools (Table 2), proffering invaluable technical guidance and a robust methodological framework for surmounting the aforementioned challenges. For detailed information, refer to Supplementary Table S2. The classic process of identifying CCIs’ patterns is as follows (Figure 2):

1. Data collection and preprocessing: Collect sequencing data such as 10× Genomics, CEL seq, Smart seq, and MARS seq from databases such as NCBI, SpatialDB, and National Genomics Data Center.

2. Data preprocessing: Apply tools such as Seurat [67], Scanpy [68], and Cell2location [69] to preprocess the downloaded sequencing data (such as quality control analysis, batch removal analysis, etc.) [17,40].

3. Annotate cell types: (1) Operate tools such as scCATCH [70], ScMap [71], and SinglR [72] to annotate cell clusters. (2) Identify cell types by combining databases such as CellMarker2 [73], Human Cell Atlas [74], and annotation of cell types [75]. (3) Manually annotate and recognize cell types. (4) Annotate cell types using existing tools or platforms, and then manually adjust cell identity [70,71,76,77].

4. Construction and prediction of CCIs’ network: Currently, the vast majority of tools for constructing communication networks rely on pre-built prior knowledge databases, and their analysis methods include co-expression analysis, network analysis, expression permutation detection, and tensor algorithms. For instance, CellPhoneDB [31] calculates the cell state specificity of ligand–receptor interactions through displacement testing, while iTALK [78] identifies highly expressed LRIs through a ranking gene list. In addition, CellCall [46] integrates ligand–receptor interactions and transcription factor (TF) activity to infer CCIs pathways, further improving the analytical methods for CCIs signaling.

Regarding the methods for predicting CCIs, existing research mainly focuses on the following three directions: (1) constructing an interaction network between CCIs signaling molecules and receptors, and using graph theory algorithms (such as PageRank) to predict CCIs relationships (patterns); (2) train machine learning models using Euclidean distance or Jaccard metric, combined with ST data, to predict the potential physical dependence between cells; (3) by combining ST data and tracking the dynamic changes in cells during development, time series models of cells (such as Markov models and ordinary differential equations (ODE)) are constructed to analyze the temporal and spatial interactions between cells [79,80,81].

5. CCIs intensity and differential analysis: After constructing a CCIs network, it is necessary to further identify the communication intensity between cells or cell autocrine. At present, the existing scoring methods include binary and continuous scoring. Binary scoring is based on expression thresholds to identify “active” or “inactive” ligand–receptor interactions. In contrast, continuous scoring calculates the product of the interacting proteins’ expression levels, offering a more nuanced measurement. However, it may be affected by the sparsity of expression data [82,83,84,85,86].

When there are multiple experimental conditions in the study, over-representation analysis (ORA) or z-score-based methods can be used to evaluate the significant differences in CCIs under different conditions, such as scDiffCom [87] and SpatialDM [88] tools can calculate CCIs differences in scRNA-seq and ST data.

6. Screening for significant LRIs: Applying existing tools to calculate communication intensity and analyze communication differences under different conditions, scoring intensity thresholds or statistical significance (such as *p*-value or adjusted *p*-value) can be set to screen for important ligand–receptor interactions, receptor–transcription factors, and metabolites, such as CellChat [89] and scFBA [90].

7. Validation of results and performance assessment of analytical tools: To corroborate decisive findings related to cell–cell interactions, communication networks, and ligand–receptor interactions dynamics, researchers can employ experimental techniques such as fluorescence in situ hybridization (FISH) and immunohistochemistry. Alternatively, machine learning models can be utilized to validate the analysis results and assess the contribution of ligand–receptor interactions to CCIs [17]. Furthermore, the performance of analytical tools can be evaluated by applying multiple tools to the same dataset. For instance, tools like LIANA [91], ESICCC [92], and LIANA+ [93] can be utilized to benchmark their efficacy and compare the biological significance of the results.

**Table 2:** CCIs analysis tools.

Tool Type	Tool Name	Note
Ligand–receptor co-expression and differential analysis	Cellphonedb [31], CellChat [89], iTALK [78], NATMI [94], CSOmap [95], NeuronChat [96], scLR [97], scConnect [98], SingleCellSignalR [37], ICELLNET [36], TraSig [99], TimiGP [100], NICHES [101], CCInx [102], Scriabin [103], ScSeqComm [104], Celltalker [105], dsCellNet [106], ScDiffCom [87], LRLoop [107], SPRUCE [108]	We will provide detailed key features and innovative points, as well as website information, for each tool in Supplementary Table S2, respectively.
Network analysis	NicheNet [34], Connectome [109], MDIC3 [110], CLARIFY [111], Giotto [26], CellAgentChat [112], cytotalk [113], exFINDER [114], SoptSC [115], SCENIC [116], ProximID [117], FlowSIg [118]	
Spatial distance and proximity analysis	SpatialCorr [119], SpaOTsc [120], BATCOM [121], COMMOT [122], SpaTalk [123], STcomm [124], SpaCET [125], CCPLS [126], CINS [127], RNA-Magnet [128], spaCI [129], SpatialDM [88], stLearn [130], MESSI [81], ScHOT [131], Squidpy [132], CellNeighborEX [133]	
Traditional machine learning and deep learning	DeepCCI [134], CytoCommunity [135], LR Hunting [136], NetPhosPan [137], GCNG [138], Neighbor-seq [139], HiVAE [140], DeepTalk [141], scMultiSim [142], RobustCCC [143], HoloNet [144], GraphComm [145], DeepLinc [146], CellEnBoost [147], ScTenifoldXct [148], SVCA [149], DIISCO [150], ISCHIA [151]	
Metabolic models and energy balance	scFBA [90], scFEA [152], COMPASS [153], MEBOCOST [154], MISTy [155]	
Tensor decomposition and matrix decomposition	ScTensor [156], Tensor-cell2cell [157], scITD [158], NCEM [159], DiSiR [160]	
TF and signaling pathway analysis	CellCall [46], CellComNet [161], CCCExplorer [83], Cell2cell [162], Commpath [163], ScMLnet [164], FunRes [165], TimeTalk [166], Domino [167], CellComm [168], DcjComm [169]	
Multiple combination methods	COMUNET [170], BulkSignalR [171], PyMINEr [172], CrossTalkeR [173], stMLnet [174]	
Comparative evaluation of cell communication tools	ESICCC/CCCbank [92], LIANA [91], LIANA+ [93]	
Analysis platform	InterCellar [175], TALKIEN [176]	
Analyze cell communication under various conditions	MOFAcell [177], DIALOGUE [178]	

## 3. Effects of CCIs on Embryonic Development

**Figure 3:**
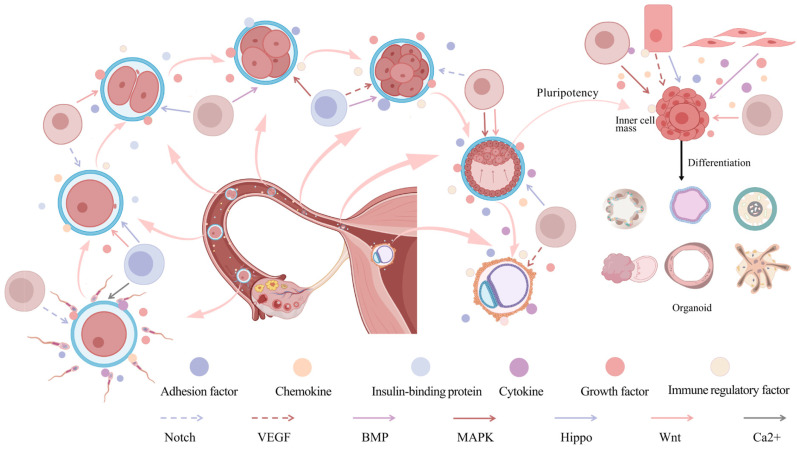
**Effects of CCIs on early embryonic development.** Early embryonic development begins with fertilization, where the sperm and egg unite to form a zygote. Through successive divisions, the embryo enters the zygotic genome activation (ZGA) stage, progresses to the blastocyst stage, and ultimately implants into the uterus. During this process, the inner cell mass retains pluripotency, under the influence of CCIs, it can differentiate into other tissues or organs. CCIs occur frequently during early embryonic development, playing a crucial role in signaling pathways by regulating small molecules and profoundly influencing developmental processes.

The CCIs network serves as the fundamental regulatory architecture in the embryonic development of complex multicellular organisms, coordinating key events in the early stages of embryogenesis and fine-tuning cellular functions at the spatial and temporal resolution at the molecular level. Multiple models of CCIs networks synergistically sustains the order and homeostasis indispensable for normal embryonic development. The highly dynamic and spatiotemporally specific regulation within the CCIs network is of paramount importance for unraveling the intricate mechanisms underlying embryonic development (Figure 3). Therefore, studying the mechanisms and regulation of CCIs is essential for understanding the fundamental processes of embryonic development and disease progression, as well as for advancing clinical research and therapeutic applications. In the following sections of this chapter, we will introduce the role of CCIs in different stages of early embryonic development and maternal fetal interaction interface.

### 3.1. Fertilization and Zygotic Genome Activation (ZGA)

The fusion of sperm and egg represents the first pivotal event orchestrated by CCIs during early embryonic development. Studies have demonstrated that, during the process of sperm–egg binding, the proteins Izumo1, Space6, and Tmem81 on the sperm surface assemble into a trimeric complex. This complex interacts specifically with Juno and CD9 proteins on the egg surface, forming a highly selective protein–protein interaction. This interaction constitutes a critical step in facilitating successful gamete fusion, ensuring the fidelity and efficiency of fertilization [179].

Following successful fertilization, CCIs continue to play a pivotal role in shaping subsequent stages of early embryonic development, particularly during the critical transition from the fertilized egg to the ZGA stage. ZGA represents a fundamental turning point in embryogenesis, signifying the comprehensive activation of the zygotic genome and the onset of autonomous regulation of gene expression by the embryo [180]. During the ZGA stage, the degradation of maternal mRNA and the transcription of zygotic genes are orchestrated by a series of intricately regulated signaling pathways. Studies have revealed that both autocrine and paracrine signals significantly influence zygotic genome activation by modulating the expression of key proteins (e.g., TGF-β family members, Dppa2/4) and transcription factors (e.g., SOX family, Oct4). These signaling molecules engage specific cell surface receptors to activate CCIs pathways such as BMP, MAPK, and Wnt, thereby initiating genomic transcription [181]. Concurrently, maternal RNA-binding proteins and small regulatory RNAs ensure that the activation of the embryonic genome occurs with precise temporal coordination. This tightly regulated interplay underscores the complexity of signaling networks required to drive embryonic development [182].

### 3.2. Blastocyst Formation and Pluripotency Maintenance

As the embryo progresses to the blastocyst stage, CCIs continue to play a pivotal role in blastocyst formation and successful implantation, marking yet another critical juncture in embryonic development. During blastocyst stage, the embryo undergoes differentiation into two distinct cell populations: the inner cell mass (ICM) and the trophectoderm (TE). Research has demonstrated that CCIs within the embryo is intricately coordinated through key signaling pathways, including Wnt, Hippo, and MAPK, which regulate blastocyst morphogenesis and establish cellular polarity. Notably, the Wnt signaling pathway is instrumental in regulating trophoblast cell differentiation, ensuring their recognition and adhesion to the endometrium, a crucial process for successful implantation [183]. Simultaneously, the Hippo signaling pathway influences the development of the ICM through paracrine signaling, driving endoderm formation and initiating the early stages of embryonic differentiation [184]. During blastocyst formation, the ICM acquires pluripotency, enabling it to differentiate into various somatic cell types. CCIs are pivotal in both the establishment of the ICM and its acquisition of pluripotency. Through direct cell contact and the secretion of signaling molecules, the ICM modulates the expression of key growth factors and cytokines, with signaling pathways such as Wnt, BMP, and VEGF collaboratively orchestrating cell differentiation and the preservation of pluripotency. Moreover, transcription factors such as Oct4, Sox2, and Nanog play indispensable roles in the differentiation process of the ICM, ensuring both its self-renewal potential and its capacity for differentiation [185,186].

### 3.3. CCIs Between Implantation and Fetus

Throughout early embryonic development, a tightly regulated network of signal transmission and dynamic CCIs operates between the maternal and fetal systems. When cells fail to interact correctly or misinterpret molecular signals, this disruption can lead to various disorders, negatively impacting both maternal health and embryonic development.

**Figure 4:**
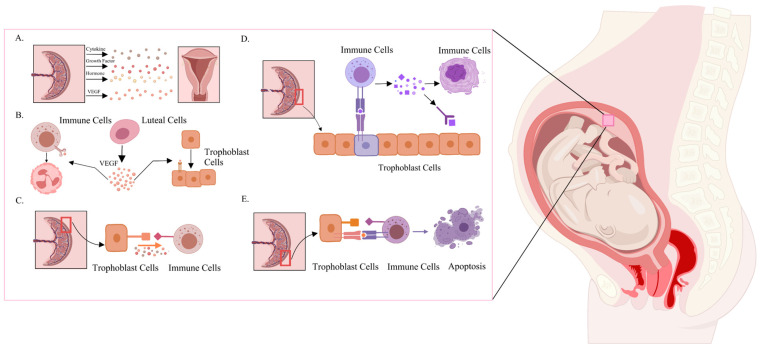
**Effects of CCIs at the mother-to-fetus binding interface.** (**A**) The placenta interacts with the endometrium by secreting small molecules, including cytokines, growth factors, hormones, and VEGF, to facilitate implantation. (**B**) At the mother-to-fetus binding interface, key CCIs involve interactions between luteal cells, immune cells, and trophoblast cells through VEGF secretion, facilitating the crosstalk between the immune system and trophoblast cells. (**C**) Trophoblast cells interact with immune cells through the secretion of small molecules. (**D**) Immune cells secrete ligands that bind to receptors on the surface of trophoblast cells, facilitating CCIs. Alternatively, they may first interact with other immune cells before engaging with trophoblast cells. (**E**) Anomalies in trophoblast-immune CCIs may lead to immune cell apoptosis.

The communication between nourishing ectodermal cells and endometrial cells during implantation is regarded as a pivotal determinant for the successful embedding of the embryo [187]. Ligands secreted by nourishing ectodermal cells, including members of the FGF and EGF families, engage with receptors on the surface of endometrial cells (such as EGFR, FGFR, and VEGFR), orchestrating critical CCIs signaling and adhesion processes that facilitate embryo implantation [188,189]. Furthermore, emerging studies have demonstrated that signaling molecules, notably VEGF and IGF, regulate endometrial angiogenesis through both autocrine and paracrine mechanisms, thereby ensuring an adequate supply of nutrients and oxygen to the developing embryo. The cascade of molecular events is essential for embryo survival and plays a crucial role in initiating early placental development, supporting continued growth post-implantation [190,191] (Figure 4A,B). The coordinated action of these signaling molecules is essential for embryonic survival and plays a fundamental role in early placental development.

In addition to angiogenesis, immune communication between the embryo and the maternal system represents another critical event during implantation. To prevent immune rejection, the maternal immune system must adapt to recognize and tolerate the semi-allogeneic embryo. Nourishing ectodermal cells engage with maternal immune cells by secreting a diverse array of regulatory factors, including cytokines from the IL family, TGF-β, and immune modulators such as the HLA, CCL, and CXCL families. These factors work synergistically to suppress excessive immune responses while maintaining the necessary immune tolerance required for successful implantation [192,193] (Figure 4C,D). What’s more, under specific conditions, nourishing ectodermal cells can actively modulate the maternal immune environment by inducing apoptosis in maternal immune cells, thereby mitigating potential immune attacks on the developing embryo [194] (Figure 4E). The results showed the pivotal role of trophoblastic ectodermal cells in establishing immune balance at the maternal–fetal interface, which is indispensable for placental formation and the subsequent normal progression of embryonic development.

## 4. Challenges and Future Directions

(1) Challenges in spatial localization and transmembrane state verification at single-cell resolution: Accurately determining the spatial localization of cells at single-cell resolution remains a significant challenge due to the absence of a standardized method. Reliance solely on sequencing data often lacks the precision required for comprehensive spatial mapping. Consequently, biological experiments are essential to validate computational predictions and enhance the reliability of findings. Addressing these limitations is particularly critical for elucidating CCIs within complex tissues. The development of robust methodologies that integrate single-cell resolution spatial localization with functional interaction analysis remains an urgent and unmet need in the field.

(2) Challenges in integrating multi-omics data: Research on CCIs patterns during embryonic development have traditionally relied on the independent analysis of scRNA-seq or ST data. While some tools, such as CCCExplorer [83], have begun incorporating bulk RNA-seq data to study CCIs within specific contexts, these approaches remain limited in scope. The emergence of multi-omics integration methodologies, however, is rapidly gaining traction as a transformative trend in the field. For instance, Sheikh BN et al. [195] utilized EMBRACE technology to isolate embryonic brain cells and combined bulk RNA-seq and scRNA-seq data to construct a comprehensive CCIs atlas during mouse embryonic brain development. This innovative approach underscored the critical role of CCIs signaling in shaping embryonic brain development. Additionally, advanced tools such as Deeptalk [141] have demonstrated the capability to integrate scRNA-seq and ST data, enabling the identification of CCIs at single-cell resolution and the tracing of cell origins within their native spatial context.

These integrative tools provide profound insights into embryonic development, cell differentiation, and disease-related immune responses, marking a significant advancement in the study of complex biological systems. Despite significant advancements, research tools capable of simultaneously integrating scRNA-seq and ST data remain scarce. In addition, existing analytical methods are often tailored to specific application scenarios, limiting their generalizability and broader applicability. Addressing these limitations, the future development of versatile and adaptive analytical tools that seamlessly integrate multi-omics data will be crucial. Such advancements will deepen the analysis of CCI networks while also improving the reliability and reproducibility of results. Ultimately, these innovations will provide a more holistic framework for understanding the complex mechanisms underlying embryonic development.

(3) Insufficient diversity modules and cross species data in databases: Currently, multiple databases related to CCIs established, but there are still significant limitations, including the following: 1. The absence of functional classification modules for autocrine and paracrine signaling pathways. 2. An insufficient representation of complex ligand–receptor interactions data. 3. A lack of visualization tools tailored to analyzing intricate communication networks. 4. Inadequate capabilities for tracking continuous cellular state transitions. 5. Limited data coverage for non-model species such as cattle, sheep, and pigs. 6. An extensive collection of CCIs data with limited precise modules dedicated to developmental biology research.

Moreover, technological constraints and gaps in prior knowledge have left many ligand–receptor interactions incompletely characterized or entirely unknown. The result underscores that building a comprehensive, dynamic, and cross species signaling molecule database is an important and challenging task, and the updating of the database is still like the hands of a clock that cannot be stopped.

(4) Insufficient interpretability of analysis results: Existing CCIs tools are mostly developed using R language >=4.1.0 or Python >= 3.7.0, and common analysis methods include the following: 1. Gene co-expression analysis based on ligand–receptor interactions and signaling molecules (such as active factors), relying on high-quality ligand–receptor interactions logarithmic databases (Table 1). The accuracy and interpretability of this method depend on the completeness and quality of the data. 2. Calculate the physical (spatial) proximity between cells using optimal transmission methods and convolutional neural network models. These methods are applicable to multiple data types and can be optimized based on data characteristics, but their disadvantages are high computational resources and time consumption, and interpretability needs to be improved.

Moving forward, the development of more efficient and interpretable analytical methodologies will provide researchers with sophisticated tools to deepen our understanding of CCIs networks [196]. Concurrently, enhancing the visualization capabilities of these tools will allow for more intuitive interpretation of analytical outcomes, thereby increasing their usability and practical application in diverse research contexts.

## 5. Discussion and Conclusions

In the wake of the precipitous accretion of high-throughput sequencing technologies, researchers are now enabled to scrutinize the elaborate processes of embryonic development from a more all-encompassing and multidimensional vantage point. Among these processes, CCIs stand as the pivotal mechanism governing embryonic development. Nonetheless, any aberrations in the communication mechanisms can potentially culminate in embryonic developmental defects and afflictions, such as congenital malformations or pregnancy-associated disorders (for instance, placental dysfunction). Consequently, the integration of multiple sequencing datasets to achieve a more profound comprehension of CCIs mechanisms has emerged as a contemporary research epicenter, particularly with respect to deciphering the mechanisms underlying cell coordination and regulation during embryonic development. This pursuit is primary to uncovering the mysteries of normal embryonic development and has profound implications for understanding the causes of developmental disorders and developing novel therapeutic strategies.

Data integration analysis has improved CCIs analysis tools change from core-gene-expression-based methods to integrate multicell features and spatial location information [105,197,198]. Combining various sequencing data allows for macroscopic study of cell composition in embryonic tissues and reveals single-cell level CCIs dynamics. This offers a new angle to understand how cells coordinate and precisely regulate functions during embryonic development.

The innovation in experimental methods has also spearheaded developmental biology research. Current techniques can analyze not just single-cell CCIs, but also pathways between multiple cell types, yielding more biologically significant insights into development. Multi-level data integration enables an understanding of developmental processes from single-cell to tissue levels. The combined use of computational and experimental methods has greatly enhanced potential in biomedical and personalized medicine fields.

Numerous studies have demonstrated that the development of datasets applicable across diverse developmental stages, along with efficient and highly interpretable analysis tools, is pivotal for conducting in-depth research into CCIs, as well as their functions in development. Simultaneously, integrating state of the art experimental techniques with tools for analyzing CCIs can further elucidate the communication mechanisms operative during development. This expands the body of knowledge in research while providing deeper and more comprehensive insights for clinical applications, effectively bridging the gap between basic research and translational medicine.

## Figures and Tables

**Table 1:** CCIs database.

Data Type	Database Name	Note
Ligand–receptor pair database	celltalkDB [29], cellphoneDB [31], KEGG [32], cellchatDB [30], Cellinker [33], NicheNet [34], Gene Ontology [35], ICELLNET [36], singlecellsignalR [37], Omnipath [38], DLRP [39], CCIDB [40], Cell-Cell Interaction Database [41], Reactome [42], connectomeDB [43], IUPHAR-DB [44], CITEdb [45], Cellcall [46], CellCommuNet [47], IUPHAR/BPS Guide to Pharmacology [48], A draft network of ligand–receptor-mediated multicellular signalling in human [49], PlantPhoneDB [50], FlyPhoneDB [51], InterCellDB [52]	We will provide detailed key features and innovative points, as well as website information, for each database in Supplementary Table S1, respectively.
Protein–protein interaction database	HPRD [53], HPMR [54], PICKLE [55], APID [56], IntAct [57], Pathway Commons [58], The Human Protein Atlas(HPA) [59], UniProt [60], STRING [61], BioGRID [62], Mapping the human membrane proteome [63], GPS-prot [64], Wiki-pi [65], iHOP [66]	
Metabolite database	MACC [28]	

## Data Availability

No datasets were generated or analyzed during the current study.

## Supplementary Materials

The following supporting information can be downloaded at https://www.mdpi.com/article/10.3390/ijms26093997/s1.

## Abbreviations

CCIs	Cell–Cell Interactions
cAMP	Cyclic Adenosine Monophosphate
GFP	Green Fluorescent Protein
HGP	Human Genome Project
RNAi	RNA Interference
bulk RNA-seq	Bulk RNA sequencing
scRNA-seq	Single Cell RNA sequencing
ST	Spatial Transcriptomics
TF	Transcription Factor
MOSTA	Mouse Organogenesis Spatiotemporal Transcriptome Atlas
ODE	Ordinary Differential Equations
ORA	Over-Representation Analysis
FISH	Fluorescence In Situ Hybridization
ZGA	Zygotic Genome Activation
ICM	Inner Cell Mass
TE	Trophectoderm

## References

[1] Grosberg R.K., Strathmann R.R. (2007). The evolution of multicellularity: A minor major transition?. Annu. Rev. Ecol. Evol. Syst..

[2] Pires-daSilva A., Sommer R.J. (2003). The evolution of signalling pathways in animal development. Nat. Rev. Genet..

[3] Singer S.J. (1992). Intercellular communication and cell-cell adhesion. Science.

[4] Huang L., Sun J., Liu B.H. (2023). Cell communication during cellular aging process. Chem. Life.

[5] Schwann T.H. (1993). Microscopial researches into the accordance in the structure and growth of animals and plants. 1847. Obes. Res..

[6] Virchow R. (1989). Cellular pathology. As based upon physiological and pathological histology. Lecture XVI—Atheromatous affection of arteries. 1858. Nutr. Rev..

[7] Piro A., Tagarelli A., Tagarelli G., Lagonia P., Quattrone A. (2008). Paul Ehrlich: The Nobel Prize in physiology or medicine 1908. Int. Rev. Immunol..

[8] Sutherland E.W., Rall T.W. (1958). Fractionation and characterization of a cyclic adenine ribonucleotide formed by tissue particles. J. Biol. Chem..

[9] Shimomura O., Johnson F.H., Saiga Y. (1962). Extraction, purification and properties of aequorin, a bioluminescent protein from the luminous hydromedusan, Aequorea. J. Cell Comp. Physiol..

[10] Krebs E.G., Fischer E.H. (1956). The phosphorylase b to a converting enzyme of rabbit skeletal muscle. Biochim. Biophys. Acta.

[11] Gilman A.G. (1987). G proteins: Transducers of receptor-generated signals. Annu. Rev. Biochem..

[12] Darnell J.E., Kerr I.M., Stark G.R. (1994). Jak-STAT pathways and transcriptional activation in response to IFNs and other extracellular signaling proteins. Science.

[13] Lander E.S., Linton L.M., Birren B., Nusbaum C., Zody M.C., Baldwin J., Devon K., Dewar K., Doyle M., FitzHugh W. (2001). Initial sequencing and analysis of the human genome. Nature.

[14] Fire A., Xu S., Montgomery M.K., Kostas S.A., Driver S.E., Mello C.C. (1998). Potent and specific genetic interference by double-stranded RNA in Caenorhabditis elegans. Nature.

[15] Tang F., Barbacioru C., Wang Y., Nordman E., Lee C., Xu N., Wang X., Bodeau J., Tuch B.B., Siddiqui A. (2009). mRNA-Seq whole-transcriptome analysis of a single cell. Nat. Methods.

[16] Ståhl P.L., Salmén F., Vickovic S., Lundmark A., Navarro J.F., Magnusson J., Giacomello S., Asp M., Westholm J.O., Huss M. (2016). Visualization and analysis of gene expression in tissue sections by spatial transcriptomics. Science.

[17] Shao X., Lu X., Liao J., Chen H., Fan X. (2020). New avenues for systematically inferring cell-cell communication: Through single-cell transcriptomics data. Protein Cell.

[18] AlJanahi A.A., Danielsen M., Dunbar C.E. (2018). An Introduction to the Analysis of Single-Cell RNA-Sequencing Data. Mol. Ther. Methods Clin. Dev..

[19] Vento-Tormo R., Efremova M., Botting R.A., Turco M.Y., Vento-Tormo M., Meyer K.B., Park J.E., Stephenson E., Polański K., Goncalves A. (2018). Single-cell reconstruction of the early maternal-fetal interface in humans. Nature.

[20] Li Z., Lin F., Zhong C.H., Wang S., Xue X., Shao Y. (2022). Single-Cell Sequencing to Unveil the Mystery of Embryonic Development. Adv. Biol..

[21] Hu X., Li J., Fu M., Zhao X., Wang W. (2021). The JAK/STAT signaling pathway: From bench to clinic. Signal Transduct. Target. Ther.

[22] Shahan R., Hsu C.W., Nolan T.M., Cole B.J., Taylor I.W., Greenstreet L., Zhang S., Afanassiev A., Vlot A.H.C., Schiebinger G. (2022). A single-cell Arabidopsis root atlas reveals developmental trajectories in wild-type and cell identity mutants. Dev. Cell.

[23] Klein A.M., Mazutis L., Akartuna I., Tallapragada N., Veres A., Li V., Peshkin L., Weitz D.A., Kirschner M.W. (2015). Droplet barcoding for single-cell transcriptomics applied to embryonic stem cells. Cell.

[24] Shalek A.K., Satija R., Shuga J., Trombetta J.J., Gennert D., Lu D., Chen P., Gertner R.S., Gaublomme J.T., Yosef N. (2014). Single-cell RNA-seq reveals dynamic paracrine control of cellular variation. Nature.

[25] Du J., Yang Y.C., An Z.J., Zhang M.H., Fu X.H., Huang Z.F., Yuan Y., Hou J. (2023). Advances in spatial transcriptomics and related data analysis strategies. J. Transl. Med..

[26] Dries R., Zhu Q., Dong R., Eng C.L., Li H., Liu K., Fu Y., Zhao T., Sarkar A., Bao F. (2021). Giotto: A toolbox for integrative analysis and visualization of spatial expression data. Genome Biol..

[27] Chen A., Liao S., Cheng M., Ma K., Wu L., Lai Y., Qiu X., Yang J., Xu J., Hao S. (2022). Spatiotemporal transcriptomic atlas of mouse organogenesis using DNA nanoball-patterned arrays. Cell.

[28] Gao J., Mo S., Wang J., Zhang M., Shi Y., Zhu C., Shang Y., Tang X., Zhang S., Wu X. (2024). MACC: A visual interactive knowledgebase of metabolite-associated cell communications. Nucleic Acids Res..

[29] Shao X., Liao J., Li C., Lu X., Cheng J., Fan X. (2021). CellTalkDB: A manually curated database of ligand-receptor interactions in humans and mice. Brief Bioinform..

[30] Jin S., Guerrero-Juarez C.F., Zhang L., Chang I., Ramos R., Kuan C.H., Myung P., Plikus M.V., Nie Q. (2021). Inference and analysis of cell-cell communication using CellChat. Nat. Commun..

[31] Efremova M., Vento-Tormo M., Teichmann S.A., Vento-Tormo R. (2020). CellPhoneDB: Inferring cell-cell communication from combined expression of multi-subunit ligand-receptor complexes. Nat. Protoc..

[32] Kanehisa M., Furumichi M., Tanabe M., Sato Y., Morishima K. (2017). KEGG: New perspectives on genomes, pathways, diseases and drugs. Nucleic Acids Res..

[33] Zhang Y., Liu T., Wang J., Zou B., Li L., Yao L., Chen K., Ning L., Wu B., Zhao X. (2021). Cellinker: A platform of ligand-receptor interactions for intercellular communication analysis. Bioinformatics.

[34] Browaeys R., Saelens W., Saeys Y. (2020). NicheNet: Modeling intercellular communication by linking ligands to target genes. Nat. Methods.

[35] Alterovitz G., Xiang M., Mohan M., Ramoni M.F. (2007). GO PaD: The Gene Ontology Partition Database. Nucleic Acids Res..

[36] Noël F., Massenet-Regad L., Carmi-Levy I., Cappuccio A., Grandclaudon M., Trichot C., Kieffer Y., Mechta-Grigoriou F., Soumelis V. (2021). Dissection of intercellular communication using the transcriptome-based framework ICELLNET. Nat. Commun..

[37] Cabello-Aguilar S., Alame M., Kon-Sun-Tack F., Fau C., Lacroix M., Colinge J. (2020). SingleCellSignalR: Inference of intercellular networks from single-cell transcriptomics. Nucleic Acids Res..

[38] Türei D., Korcsmáros T., Saez-Rodriguez J. (2016). OmniPath: Guidelines and gateway for literature-curated signaling pathway resources. Nat. Methods.

[39] Graeber T.G., Eisenberg D. (2001). Bioinformatic identification of potential autocrine signaling loops in cancers from gene expression profiles. Nat. Genet..

[40] Noh J.Y., Lee H.I., Choi J.H., Cho S.H., Yi Y.H., Lim J.H., Myung E.B., Shin Y.J., Shin H.J., Woo H.G. (2023). CCIDB: A manually curated cell-cell interaction database with cell context information. Database.

[41] Isserlin R., Voisin V., Ailles L., Bader G.D. (2020). Cell-Cell Interaction Database. Zenodo.

[42] Gillespie M., Jassal B., Stephan R., Milacic M., Rothfels K., Senff-Ribeiro A., Griss J., Sevilla C., Matthews L., Gong C. (2022). The reactome pathway knowledgebase 2022. Nucleic Acids Res..

[43] Hodge M.R., Horton W., Brown T., Herrick R., Olsen T., Hileman M.E., McKay M., Archie K.A., Cler E., Harms M.P. (2016). ConnectomeDB--Sharing human brain connectivity data. Neuroimage.

[44] Sharman J.L., Mpamhanga C.P., Spedding M., Germain P., Staels B., Dacquet C., Laudet V., Harmar A.J. (2011). IUPHAR-DB: New receptors and tools for easy searching and visualization of pharmacological data. Nucleic Acids Res..

[45] Shan N., Lu Y., Guo H., Li D., Jiang J., Yan L., Gao J., Ren Y., Zhao X., Hou L. (2022). CITEdb: A manually curated database of cell-cell interactions in human. Bioinformatics.

[46] Zhang Y., Liu T., Hu X., Wang M., Wang J., Zou B., Tan P., Cui T., Dou Y., Ning L. (2021). CellCall: Integrating paired ligand-receptor and transcription factor activities for cell-cell communication. Nucleic Acids Res..

[47] Ma Q., Li Q., Zheng X., Pan J. (2024). CellCommuNet: An atlas of cell-cell communication networks from single-cell RNA sequencing of human and mouse tissues in normal and disease states. Nucleic Acids Res..

[48] Pawson A.J., Sharman J.L., Benson H.E., Faccenda E., Alexander S.P., Buneman O.P., Davenport A.P., McGrath J.C., Peters J.A., Southan C. (2014). The IUPHAR/BPS Guide to PHARMACOLOGY: An expert-driven knowledgebase of drug targets and their ligands. Nucleic Acids Res..

[49] Ramilowski J.A., Goldberg T., Harshbarger J., Kloppmann E., Lizio M., Satagopam V.P., Itoh M., Kawaji H., Carninci P., Rost B. (2015). A draft network of ligand-receptor-mediated multicellular signalling in human. Nat. Commun..

[50] Xu C., Ma D., Ding Q., Zhou Y., Zheng H.L. (2022). PlantPhoneDB: A manually curated pan-plant database of ligand-receptor pairs infers cell-cell communication. Plant Biotechnol. J..

[51] Liu Y., Li J.S.S., Rodiger J., Comjean A., Attrill H., Antonazzo G., Brown N.H., Hu Y., Perrimon N. (2022). FlyPhoneDB: An integrated web-based resource for cell-cell communication prediction in Drosophila. Genetics.

[52] Jin Z., Zhang X., Dai X., Huang J., Hu X., Zhang J., Shi L. (2022). InterCellDB: A User-Defined Database for Inferring Intercellular Networks. Adv. Sci..

[53] Keshava Prasad T.S., Goel R., Kandasamy K., Keerthikumar S., Kumar S., Mathivanan S., Telikicherla D., Raju R., Shafreen B., Venugopal A. (2009). Human Protein Reference Database—2009 update. Nucleic Acids Res..

[54] Ben-Shlomo I., Yu Hsu S., Rauch R., Kowalski H.W., Hsueh A.J. (2003). Signaling receptome: A genomic and evolutionary perspective of plasma membrane receptors involved in signal transduction. Sci. STKE.

[55] Dimitrakopoulos G.N., Klapa M.I., Moschonas N.K. (2021). PICKLE 3.0: Enriching the human meta-database with the mouse protein interactome extended via mouse-human orthology. Bioinformatics.

[56] Alonso-López D., Campos-Laborie F.J., Gutiérrez M.A., Lambourne L., Calderwood M.A., Vidal M., De Las Rivas J. (2019). APID database: Redefining protein-protein interaction experimental evidences and binary interactomes. Database.

[57] Aranda B., Achuthan P., Alam-Faruque Y., Armean I., Bridge A., Derow C., Feuermann M., Ghanbarian A.T., Kerrien S., Khadake J. (2010). The IntAct molecular interaction database in 2010. Nucleic Acids Res..

[58] Rodchenkov I., Babur O., Luna A., Aksoy B.A., Wong J.V., Fong D., Franz M., Siper M.C., Cheung M., Wrana M. (2020). Pathway Commons 2019 Update: Integration, analysis and exploration of pathway data. Nucleic Acids Res..

[59] Thul P.J., Lindskog C. (2018). The human protein atlas: A spatial map of the human proteome. Protein Sci..

[60] (2021). UniProt: The universal protein knowledgebase in 2021. Nucleic Acids Res..

[61] Szklarczyk D., Franceschini A., Kuhn M., Simonovic M., Roth A., Minguez P., Doerks T., Stark M., Muller J., Bork P. (2011). The STRING database in 2011: Functional interaction networks of proteins, globally integrated and scored. Nucleic Acids Res..

[62] Stark C., Breitkreutz B.J., Chatr-Aryamontri A., Boucher L., Oughtred R., Livstone M.S., Nixon J., Van Auken K., Wang X., Shi X. (2011). The BioGRID Interaction Database: 2011 update. Nucleic Acids Res..

[63] Almén M.S., Nordström K.J., Fredriksson R., Schiöth H.B. (2009). Mapping the human membrane proteome: A majority of the human membrane proteins can be classified according to function and evolutionary origin. BMC Biol..

[64] Fahey M.E., Bennett M.J., Mahon C., Jäger S., Pache L., Kumar D., Shapiro A., Rao K., Chanda S.K., Craik C.S. (2011). GPS-Prot: A web-based visualization platform for integrating host-pathogen interaction data. BMC Bioinform..

[65] Orii N., Ganapathiraju M.K. (2012). Wiki-pi: A web-server of annotated human protein-protein interactions to aid in discovery of protein function. PLoS ONE.

[66] Fernández J.M., Hoffmann R., Valencia A. (2007). iHOP web services. Nucleic Acids Res..

[67] Hao Y., Stuart T., Kowalski M.H., Choudhary S., Hoffman P., Hartman A., Srivastava A., Molla G., Madad S., Fernandez-Granda C. (2024). Dictionary learning for integrative, multimodal and scalable single-cell analysis. Nat. Biotechnol..

[68] Wolf F.A., Angerer P., Theis F.J. (2018). SCANPY: Large-scale single-cell gene expression data analysis. Genome Biol..

[69] Kleshchevnikov V., Shmatko A., Dann E., Aivazidis A., King H.W., Li T., Elmentaite R., Lomakin A., Kedlian V., Gayoso A. (2022). Cell2location maps fine-grained cell types in spatial transcriptomics. Nat. Biotechnol..

[70] Shao X., Liao J., Lu X., Xue R., Ai N., Fan X. (2020). scCATCH: Automatic Annotation on Cell Types of Clusters from Single-Cell RNA Sequencing Data. iScience.

[71] Kiselev V.Y., Yiu A., Hemberg M. (2018). scmap: Projection of single-cell RNA-seq data across data sets. Nat. Methods.

[72] Aran D., Looney A.P., Liu L., Wu E., Fong V., Hsu A., Chak S., Naikawadi R.P., Wolters P.J., Abate A.R. (2019). Reference-based analysis of lung single-cell sequencing reveals a transitional profibrotic macrophage. Nat. Immunol..

[73] Hu C., Li T., Xu Y., Zhang X., Li F., Bai J., Chen J., Jiang W., Yang K., Ou Q. (2023). CellMarker 2.0: An updated database of manually curated cell markers in human/mouse and web tools based on scRNA-seq data. Nucleic Acids Res..

[74] Regev A., Teichmann S.A., Lander E.S., Amit I., Benoist C., Birney E., Bodenmiller B., Campbell P., Carninci P., Clatworthy M. (2017). The Human Cell Atlas. Elife.

[75] Quan F., Liang X., Cheng M., Yang H., Liu K., He S., Sun S., Deng M., He Y., Liu W. (2023). Annotation of cell types (ACT): A convenient web server for cell type annotation. Genome Med..

[76] Macosko E.Z., Basu A., Satija R., Nemesh J., Shekhar K., Goldman M., Tirosh I., Bialas A.R., Kamitaki N., Martersteck E.M. (2015). Highly Parallel Genome-wide Expression Profiling of Individual Cells Using Nanoliter Droplets. Cell.

[77] Zheng L., Liang P., Long C., Li H., Li H., Liang Y., He X., Xi Q., Xing Y., Zuo Y. (2023). EmAtlas: A comprehensive atlas for exploring spatiotemporal activation in mammalian embryogenesis. Nucleic Acids Res..

[78] Wang Y., Wang R., Zhang S., Song S., Jiang C., Han G., Wang M., Ajani J., Futreal A., Wang L. (2019). iTALK: An R Package to Characterize and Illustrate Intercellular Communication. bioRxiv.

[79] Satija R., Farrell J.A., Gennert D., Schier A.F., Regev A. (2015). Spatial reconstruction of single-cell gene expression data. Nat. Biotechnol..

[80] Navarro J.F., Sjöstrand J., Salmén F., Lundeberg J., Ståhl P.L. (2017). ST Pipeline: An automated pipeline for spatial mapping of unique transcripts. Bioinformatics.

[81] Li D., Ding J., Bar-Joseph Z. (2021). Identifying signaling genes in spatial single-cell expression data. Bioinformatics.

[82] Armingol E., Officer A., Harismendy O., Lewis N.E. (2021). Deciphering cell-cell interactions and communication from gene expression. Nat. Rev. Genet..

[83] Choi H., Sheng J., Gao D., Li F., Durrans A., Ryu S., Lee S.B., Narula N., Rafii S., Elemento O. (2015). Transcriptome analysis of individual stromal cell populations identifies stroma-tumor crosstalk in mouse lung cancer model. Cell Rep..

[84] Costa-Silva J., Domingues D., Lopes F.M. (2017). RNA-Seq differential expression analysis: An extended review and a software tool. PLoS ONE.

[85] Wang T., Li B., Nelson C.E., Nabavi S. (2019). Comparative analysis of differential gene expression analysis tools for single-cell RNA sequencing data. BMC Bioinform..

[86] Kumar M.P., Du J., Lagoudas G., Jiao Y., Sawyer A., Drummond D.C., Lauffenburger D.A., Raue A. (2018). Analysis of Single-Cell RNA-Seq Identifies Cell-Cell Communication Associated with Tumor Characteristics. Cell Rep..

[87] Lagger C., Ursu E., Equey A., Avelar R.A., Pisco A.O., Tacutu R., de Magalhães J.P. (2023). scDiffCom: A tool for differential analysis of cell-cell interactions provides a mouse atlas of aging changes in intercellular communication. Nat. Aging.

[88] Li Z., Wang T., Liu P., Huang Y. (2023). SpatialDM for rapid identification of spatially co-expressed ligand-receptor and revealing cell-cell communication patterns. Nat. Commun..

[89] Jin S., Plikus M.V., Nie Q. (2024). CellChat for systematic analysis of cell-cell communication from single-cell transcriptomics. Nat. Protoc..

[90] Damiani C., Maspero D., Di Filippo M., Colombo R., Pescini D., Graudenzi A., Westerhoff H.V., Alberghina L., Vanoni M., Mauri G. (2019). Integration of single-cell RNA-seq data into population models to characterize cancer metabolism. PLoS Comput. Biol..

[91] Dimitrov D., Türei D., Garrido-Rodriguez M., Burmedi P.L., Nagai J.S., Boys C., Ramirez Flores R.O., Kim H., Szalai B., Costa I.G. (2022). Comparison of methods and resources for cell-cell communication inference from single-cell RNA-Seq data. Nat. Commun..

[92] Luo J., Deng M., Zhang X., Sun X. (2023). ESICCC as a systematic computational framework for evaluation, selection, and integration of cell-cell communication inference methods. Genome Res..

[93] Dimitrov D., Schäfer P.S.L., Farr E., Rodriguez-Mier P., Lobentanzer S., Badia I.M.P., Dugourd A., Tanevski J., Ramirez Flores R.O., Saez-Rodriguez J. (2024). LIANA+ provides an all-in-one framework for cell-cell communication inference. Nat. Cell Biol..

[94] Hou R., Denisenko E., Ong H.T., Ramilowski J.A., Forrest A.R.R. (2020). Predicting cell-to-cell communication networks using NATMI. Nat. Commun..

[95] Ren X., Zhong G., Zhang Q., Zhang L., Sun Y., Zhang Z. (2020). Reconstruction of cell spatial organization from single-cell RNA sequencing data based on ligand-receptor mediated self-assembly. Cell Res..

[96] Zhao W., Johnston K.G., Ren H., Xu X., Nie Q. (2023). Inferring neuron-neuron communications from single-cell transcriptomics through NeuronChat. Nat. Commun..

[97] Liu Q., Hsu C.Y., Li J., Shyr Y. (2022). Dysregulated ligand-receptor interactions from single-cell transcriptomics. Bioinformatics.

[98] Jakobsson J.E.T., Spjuth O., Lagerström M.C. (2021). scConnect: A method for exploratory analysis of cell-cell communication based on single-cell RNA-sequencing data. Bioinformatics.

[99] Li D., Velazquez J.J., Ding J., Hislop J., Ebrahimkhani M.R., Bar-Joseph Z. (2022). TraSig: Inferring cell-cell interactions from pseudotime ordering of scRNA-Seq data. Genome Biol..

[100] Li C., Zhang B., Schaafsma E., Reuben A., Wang L., Turk M.J., Zhang J., Cheng C. (2023). TimiGP: Inferring cell-cell interactions and prognostic associations in the tumor immune microenvironment through gene pairs. Cell Rep. Med..

[101] Raredon M.S.B., Yang J., Kothapalli N., Lewis W., Kaminski N., Niklason L.E., Kluger Y. (2023). Comprehensive visualization of cell-cell interactions in single-cell and spatial transcriptomics with NICHES. Bioinformatics.

[102] Ximerakis M., Lipnick S.L., Innes B.T., Simmons S.K., Adiconis X., Dionne D., Mayweather B.A., Nguyen L., Niziolek Z., Ozek C. (2019). Single-cell transcriptomic profiling of the aging mouse brain. Nat. Neurosci..

[103] Wilk A.J., Shalek A.K., Holmes S., Blish C.A. (2024). Comparative analysis of cell-cell communication at single-cell resolution. Nat. Biotechnol..

[104] Baruzzo G., Cesaro G., Di Camillo B. (2022). Identify, quantify and characterize cellular communication from single-cell RNA sequencing data with scSeqComm. Bioinformatics.

[105] Vahid M.R., Brown E.L., Steen C.B., Zhang W., Jeon H.S., Kang M., Gentles A.J., Newman A.M. (2023). High-resolution alignment of single-cell and spatial transcriptomes with CytoSPACE. Nat. Biotechnol..

[106] Song Z., Wang T., Wu Y., Fan M., Wu H. (2022). dsCellNet: A new computational tool to infer cell-cell communication networks in the developing and aging brain. Comput. Struct. Biotechnol. J..

[107] Xin Y., Lyu P., Jiang J., Zhou F., Wang J., Blackshaw S., Qian J. (2022). LRLoop: A method to predict feedback loops in cell-cell communication. Bioinformatics.

[108] Subedi S., Park Y.P. (2023). Single-cell pair-wise relationships untangled by composite embedding model. iScience.

[109] Raredon M.S.B., Yang J., Garritano J., Wang M., Kushnir D., Schupp J.C., Adams T.S., Greaney A.M., Leiby K.L., Kaminski N. (2022). Computation and visualization of cell-cell signaling topologies in single-cell systems data using Connectome. Sci. Rep..

[110] Liu Y., Zhang Y., Chang X., Liu X. (2024). MDIC3: Matrix decomposition to infer cell-cell communication. Patterns.

[111] Bafna M., Li H., Zhang X. (2023). CLARIFY: Cell-cell interaction and gene regulatory network refinement from spatially resolved transcriptomics. Bioinformatics.

[112] Raghavan V., Li Y., Ding J. (2024). Harnessing Agent-Based Modeling in CellAgentChat to Unravel Cell-Cell Interactions from Single-Cell Data. bioRxiv.

[113] Hu Y., Peng T., Gao L., Tan K. (2021). CytoTalk: De novo construction of signal transduction networks using single-cell transcriptomic data. Sci. Adv..

[114] He C., Zhou P., Nie Q. (2023). exFINDER: Identify external communication signals using single-cell transcriptomics data. Nucleic Acids Res..

[115] Wang S., Karikomi M., MacLean A.L., Nie Q. (2019). Cell lineage and communication network inference via optimization for single-cell transcriptomics. Nucleic Acids Res..

[116] Aibar S., González-Blas C.B., Moerman T., Huynh-Thu V.A., Imrichova H., Hulselmans G., Rambow F., Marine J.C., Geurts P., Aerts J. (2017). SCENIC: Single-cell regulatory network inference and clustering. Nat. Methods.

[117] Boisset J.C., Vivié J., Grün D., Muraro M.J., Lyubimova A., van Oudenaarden A. (2018). Mapping the physical network of cellular interactions. Nat. Methods.

[118] Almet A.A., Tsai Y.C., Watanabe M., Nie Q. (2024). Inferring pattern-driving intercellular flows from single-cell and spatial transcriptomics. Nat. Methods.

[119] Bernstein M.N., Ni Z., Prasad A., Brown J., Mohanty C., Stewart R., Newton M.A., Kendziorski C. (2022). SpatialCorr identifies gene sets with spatially varying correlation structure. Cell Rep. Methods.

[120] Cang Z., Nie Q. (2020). Inferring spatial and signaling relationships between cells from single cell transcriptomic data. Nat. Commun..

[121] Wu D., Gaskins J.T., Sekula M., Datta S. (2023). Inferring Cell-Cell Communications from Spatially Resolved Transcriptomics Data Using a Bayesian Tweedie Model. Genes.

[122] Cang Z., Zhao Y., Almet A.A., Stabell A., Ramos R., Plikus M.V., Atwood S.X., Nie Q. (2023). Screening cell-cell communication in spatial transcriptomics via collective optimal transport. Nat. Methods.

[123] Shao X., Li C., Yang H., Lu X., Liao J., Qian J., Wang K., Cheng J., Yang P., Chen H. (2022). Knowledge-graph-based cell-cell communication inference for spatially resolved transcriptomic data with SpaTalk. Nat. Commun..

[124] Qu F., Li W., Xu J., Zhang R., Ke J., Ren X., Meng X., Qin L., Zhang J., Lu F. (2023). Three-dimensional molecular architecture of mouse organogenesis. Nat. Commun..

[125] Ru B., Huang J., Zhang Y., Aldape K., Jiang P. (2023). Estimation of cell lineages in tumors from spatial transcriptomics data. Nat. Commun..

[126] Tsuchiya T., Hori H., Ozaki H. (2022). CCPLS reveals cell-type-specific spatial dependence of transcriptomes in single cells. Bioinformatics.

[127] Yuan Y., Cosme C., Adams T.S., Schupp J., Sakamoto K., Xylourgidis N., Ruffalo M., Li J., Kaminski N., Bar-Joseph Z. (2022). CINS: Cell Interaction Network inference from Single cell expression data. PLoS Comput. Biol..

[128] Baccin C., Al-Sabah J., Velten L., Helbling P.M., Grünschläger F., Hernández-Malmierca P., Nombela-Arrieta C., Steinmetz L.M., Trumpp A., Haas S. (2020). Combined single-cell and spatial transcriptomics reveal the molecular, cellular and spatial bone marrow niche organization. Nat. Cell Biol..

[129] Tang Z., Zhang T., Yang B., Su J., Song Q. (2023). spaCI: Deciphering spatial cellular communications through adaptive graph model. Brief Bioinform..

[130] Pham D., Tan X., Balderson B., Xu J., Grice L.F., Yoon S., Willis E.F., Tran M., Lam P.Y., Raghubar A. (2023). Robust mapping of spatiotemporal trajectories and cell-cell interactions in healthy and diseased tissues. Nat. Commun..

[131] Ghazanfar S., Lin Y., Su X., Lin D.M., Patrick E., Han Z.G., Marioni J.C., Yang J.Y.H. (2020). Investigating higher-order interactions in single-cell data with scHOT. Nat. Methods.

[132] Palla G., Spitzer H., Klein M., Fischer D., Schaar A.C., Kuemmerle L.B., Rybakov S., Ibarra I.L., Holmberg O., Virshup I. (2022). Squidpy: A scalable framework for spatial omics analysis. Nat. Methods.

[133] Kim H., Kumar A., Lövkvist C., Palma A.M., Martin P., Kim J., Bhoopathi P., Trevino J., Fisher P., Madan E. (2023). CellNeighborEX: Deciphering neighbor-dependent gene expression from spatial transcriptomics data. Mol. Syst. Biol..

[134] Yang W., Wang P., Luo M., Cai Y., Xu C., Xue G., Jin X., Cheng R., Que J., Pang F. (2023). DeepCCI: A deep learning framework for identifying cell-cell interactions from single-cell RNA sequencing data. Bioinformatics.

[135] Hu Y., Rong J., Xu Y., Xie R., Peng J., Gao L., Tan K. (2024). Unsupervised and supervised discovery of tissue cellular neighborhoods from cell phenotypes. Nat. Methods.

[136] Lu M., Sha Y., Silva T.C., Colaprico A., Sun X., Ban Y., Wang L., Lehmann B.D., Chen X.S. (2021). LR Hunting: A Random Forest Based Cell-Cell Interaction Discovery Method for Single-Cell Gene Expression Data. Front. Genet..

[137] Fenoy E., Izarzugaza J.M.G., Jurtz V., Brunak S., Nielsen M. (2019). A generic deep convolutional neural network framework for prediction of receptor-ligand interactions-NetPhosPan: Application to kinase phosphorylation prediction. Bioinformatics.

[138] Yuan Y., Bar-Joseph Z. (2020). GCNG: Graph convolutional networks for inferring gene interaction from spatial transcriptomics data. Genome Biol..

[139] Ghaddar B., De S. (2022). Reconstructing physical cell interaction networks from single-cell data using Neighbor-seq. Nucleic Acids Res..

[140] Liu S., Zhang Y., Peng J., Shang X. (2024). An improved hierarchical variational autoencoder for cell-cell communication estimation using single-cell RNA-seq data. Brief Funct. Genom..

[141] Yang W., Wang P., Xu S., Wang T., Luo M., Cai Y., Xu C., Xue G., Que J., Ding Q. (2024). Deciphering cell-cell communication at single-cell resolution for spatial transcriptomics with subgraph-based graph attention network. Nat. Commun..

[142] Li H., Zhang Z., Squires M., Chen X., Zhang X. (2023). scMultiSim: Simulation of multi-modality single cell data guided by cell-cell interactions and gene regulatory networks. Res. Sq..

[143] Zhang C., Gao L., Hu Y., Huang Z. (2023). RobustCCC: A robustness evaluation tool for cell-cell communication methods. Front. Genet..

[144] Li H., Ma T., Hao M., Guo W., Gu J., Zhang X., Wei L. (2023). Decoding functional cell-cell communication events by multi-view graph learning on spatial transcriptomics. Brief Bioinform..

[145] So E., Hayat S., Nair S.K., Wang B., Haibe-Kains B. (2024). GraphComm: A Graph-based Deep Learning Method to Predict Cell-Cell Communication in single-cell RNAseq data. bioRxiv.

[146] Li R., Yang X. (2022). De novo reconstruction of cell interaction landscapes from single-cell spatial transcriptome data with DeepLinc. Genome Biol..

[147] Peng L., Yuan R., Han C., Han G., Tan J., Wang Z., Chen M., Chen X. (2023). CellEnBoost: A Boosting-Based Ligand-Receptor Interaction Identification Model for Cell-to-Cell Communication Inference. IEEE Trans. Nanobiosci..

[148] Yang Y., Li G., Zhong Y., Xu Q., Lin Y.T., Roman-Vicharra C., Chapkin R.S., Cai J.J. (2023). scTenifoldXct: A semi-supervised method for predicting cell-cell interactions and mapping cellular communication graphs. Cell Syst..

[149] Arnol D., Schapiro D., Bodenmiller B., Saez-Rodriguez J., Stegle O. (2019). Modeling Cell-Cell Interactions from Spatial Molecular Data with Spatial Variance Component Analysis. Cell Rep..

[150] Park C., Mani S., Beltran-Velez N., Maurer K., Huang T., Li S., Gohil S., Livak K.J., Knowles D.A., Wu C.J. (2024). A Bayesian framework for inferring dynamic intercellular interactions from time-series single-cell data. Genome Res..

[151] Lafzi A., Borrelli C., Baghai Sain S., Bach K., Kretz J.A., Handler K., Regan-Komito D., Ficht X., Frei A., Moor A. (2024). Identifying Spatial Co-occurrence in Healthy and InflAmed tissues (ISCHIA). Mol. Syst. Biol..

[152] Alghamdi N., Chang W., Dang P., Lu X., Wan C., Gampala S., Huang Z., Wang J., Ma Q., Zang Y. (2021). A graph neural network model to estimate cell-wise metabolic flux using single-cell RNA-seq data. Genome Res..

[153] Wagner A., Wang C., Fessler J., DeTomaso D., Avila-Pacheco J., Kaminski J., Zaghouani S., Christian E., Thakore P., Schellhaass B. (2021). Metabolic modeling of single Th17 cells reveals regulators of autoimmunity. Cell.

[154] Cui K., Gao X., Wang B., Wu H., Arulsamy K., Dong Y., Xiao Y., Jiang X., Malovichko M.V., Li K. (2023). Epsin Nanotherapy Regulates Cholesterol Transport to Fortify Atheroma Regression. Circ. Res..

[155] Tanevski J., Flores R.O.R., Gabor A., Schapiro D., Saez-Rodriguez J. (2022). Explainable multiview framework for dissecting spatial relationships from highly multiplexed data. Genome Biol..

[156] Tsuyuzaki K., Ishii M., Nikaido I. (2023). Sctensor detects many-to-many cell-cell interactions from single cell RNA-sequencing data. BMC Bioinform..

[157] Armingol E., Baghdassarian H.M., Martino C., Perez-Lopez A., Aamodt C., Knight R., Lewis N.E. (2022). Context-aware deconvolution of cell-cell communication with Tensor-cell2cell. Nat. Commun..

[158] Mitchel J., Gordon M.G., Perez R.K., Biederstedt E., Bueno R., Ye C.J., Kharchenko P.V. (2024). Coordinated, multicellular patterns of transcriptional variation that stratify patient cohorts are revealed by tensor decomposition. Nat. Biotechnol..

[159] Fischer D.S., Schaar A.C., Theis F.J. (2023). Modeling intercellular communication in tissues using spatial graphs of cells. Nat. Biotechnol..

[160] Lenczner G., Le Saux B., Luminari N., Chan-Hon-Tong A., Le Besnerais G. (2020). DISIR: Deep image segmentation with interactive refinement. arXiv.

[161] Peng L., Tan J., Xiong W., Zhang L., Wang Z., Yuan R., Li Z., Chen X. (2023). Deciphering ligand-receptor-mediated intercellular communication based on ensemble deep learning and the joint scoring strategy from single-cell transcriptomic data. Comput. Biol. Med..

[162] Armingol E., Ghaddar A., Joshi C.J., Baghdassarian H., Shamie I., Chan J., Her H.L., Berhanu S., Dar A., Rodriguez-Armstrong F. (2022). Inferring a spatial code of cell-cell interactions across a whole animal body. PLoS Comput. Biol..

[163] Lu H., Ping J., Zhou G., Zhao Z., Gao W., Jiang Y., Quan C., Lu Y., Zhou G. (2022). CommPath: An R package for inference and analysis of pathway-mediated cell-cell communication chain from single-cell transcriptomics. Comput. Struct. Biotechnol. J..

[164] Cheng J., Zhang J., Wu Z., Sun X. (2021). Inferring microenvironmental regulation of gene expression from single-cell RNA sequencing data using scMLnet with an application to COVID-19. Brief Bioinform..

[165] Jung S., Singh K., Del Sol A. (2021). FunRes: Resolving tissue-specific functional cell states based on a cell-cell communication network model. Brief Bioinform..

[166] Wang L., Zheng Y., Sun Y., Mao S., Li H., Bo X., Li C., Chen H. (2023). TimeTalk uses single-cell RNA-seq datasets to decipher cell-cell communication during early embryo development. Commun. Biol..

[167] Cherry C., Maestas D.R., Han J., Andorko J.I., Cahan P., Fertig E.J., Garmire L.X., Elisseeff J.H. (2021). Computational reconstruction of the signalling networks surrounding implanted biomaterials from single-cell transcriptomics. Nat. Biomed. Eng..

[168] Lummertz da Rocha E., Kubaczka C., Sugden W.W., Najia M.A., Jing R., Markel A., LeBlanc Z.C., Dos Santos Peixoto R., Falchetti M., Collins J.J. (2022). CellComm infers cellular crosstalk that drives haematopoietic stem and progenitor cell development. Nat. Cell Biol..

[169] Ding Q., Yang W., Xue G., Liu H., Cai Y., Que J., Jin X., Luo M., Pang F., Yang Y. (2024). Dimension reduction, cell clustering, and cell-cell communication inference for single-cell transcriptomics with DcjComm. Genome Biol..

[170] Solovey M., Scialdone A. (2020). COMUNET: A tool to explore and visualize intercellular communication. Bioinformatics.

[171] Villemin J.P., Bassaganyas L., Pourquier D., Boissière F., Cabello-Aguilar S., Crapez E., Tanos R., Cornillot E., Turtoi A., Colinge J. (2023). Inferring ligand-receptor cellular networks from bulk and spatial transcriptomic datasets with BulkSignalR. Nucleic Acids Res..

[172] Tyler S.R., Rotti P.G., Sun X., Yi Y., Xie W., Winter M.C., Flamme-Wiese M.J., Tucker B.A., Mullins R.F., Norris A.W. (2019). PyMINEr Finds Gene and Autocrine-Paracrine Networks from Human Islet scRNA-Seq. Cell Rep..

[173] Nagai J.S., Leimkühler N.B., Schaub M.T., Schneider R.K., Costa I.G. (2021). CrossTalkeR: Analysis and visualization of ligand-receptorne tworks. Bioinformatics.

[174] Cheng J., Yan L., Nie Q., Sun X. (2023). Modeling and inference of spatial intercellular communications and multilayer signaling regulations using stMLnet. bioRxiv.

[175] Interlandi M., Kerl K., Dugas M. (2022). InterCellar enables interactive analysis and exploration of cell-cell communication in single-cell transcriptomic data. Commun. Biol..

[176] Moratalla-Navarro F., Moreno V., Sanz-Pamplona R. (2023). TALKIEN: crossTALK IntEraction Network. A web-based tool for deciphering molecular communication through ligand-receptor interactions. Mol. Omics.

[177] Ramirez Flores R.O., Lanzer J.D., Dimitrov D., Velten B., Saez-Rodriguez J. (2023). Multicellular factor analysis of single-cell data for a tissue-centric understanding of disease. Elife.

[178] Jerby-Arnon L., Regev A. (2022). DIALOGUE maps multicellular programs in tissue from single-cell or spatial transcriptomics data. Nat. Biotechnol..

[179] Deneke V.E., Blaha A., Lu Y., Suwita J.P., Draper J.M., Phan C.S., Panser K., Schleiffer A., Jacob L., Humer T. (2024). A conserved fertilization complex bridges sperm and egg in vertebrates. Cell.

[180] Liu B., Xu Q., Wang Q., Feng S., Lai F., Wang P., Zheng F., Xiang Y., Wu J., Nie J. (2020). The landscape of RNA Pol II binding reveals a stepwise transition during ZGA. Nature.

[181] Li H., Long C., Xiang J., Liang P., Li X., Zuo Y. (2021). Dppa2/4 as a trigger of signaling pathways to promote zygote genome activation by binding to CG-rich region. Brief Bioinform..

[182] Vastenhouw N.L., Cao W.X., Lipshitz H.D. (2019). The maternal-to-zygotic transition revisited. Development.

[183] Dietrich B., Haider S., Meinhardt G., Pollheimer J., Knöfler M. (2022). WNT and NOTCH signaling in human trophoblast development and differentiation. Cell Mol. Life Sci..

[184] Wu Z., Guan K.L. (2021). Hippo Signaling in Embryogenesis and Development. Trends Biochem. Sci..

[185] Li L., Lai F., Hu X., Liu B., Lu X., Lin Z., Liu L., Xiang Y., Frum T., Halbisen M.A. (2023). Multifaceted SOX2-chromatin interaction underpins pluripotency progression in early embryos. Science.

[186] Lai F., Li L., Hu X., Liu B., Zhu Z., Liu L., Fan Q., Tian H., Xu K., Lu X. (2023). NR5A2 connects zygotic genome activation to the first lineage segregation in totipotent embryos. Cell Res..

[187] Suryawanshi H., Morozov P., Straus A., Sahasrabudhe N., Max K.E.A., Garzia A., Kustagi M., Tuschl T., Williams Z. (2018). A single-cell survey of the human first-trimester placenta and decidua. Sci. Adv..

[188] Ruane P.T., Garner T., Parsons L., Babbington P.A., Wangsaputra I., Kimber S.J., Stevens A., Westwood M., Brison D.R., Aplin J.D. (2022). Trophectoderm differentiation to invasive syncytiotrophoblast is promoted by endometrial epithelial cells during human embryo implantation. Hum. Reprod..

[189] Barrientos G., Freitag N., Tirado-González I., Unverdorben L., Jeschke U., Thijssen V.L., Blois S.M. (2014). Involvement of galectin-1 in reproduction: Past, present and future. Hum. Reprod. Update.

[190] Nowak D.G., Amin E.M., Rennel E.S., Hoareau-Aveilla C., Gammons M., Damodoran G., Hagiwara M., Harper S.J., Woolard J., Ladomery M.R. (2010). Regulation of vascular endothelial growth factor (VEGF) splicing from pro-angiogenic to anti-angiogenic isoforms: A novel therapeutic strategy for angiogenesis. J. Biol. Chem..

[191] Lin S., Zhang Q., Shao X., Zhang T., Xue C., Shi S., Zhao D., Lin Y. (2017). IGF-1 promotes angiogenesis in endothelial cells/adipose-derived stem cells co-culture system with activation of PI3K/Akt signal pathway. Cell Prolif..

[192] Duan L., Zhang X.D., Miao W.Y., Sun Y.J., Xiong G., Wu Q., Li G., Yang P., Yu H., Li H. (2018). PDGFRβ Cells Rapidly Relay Inflammatory Signal from the Circulatory System to Neurons via Chemokine CCL2. Neuron.

[193] Yang M., Ong J., Meng F., Zhang F., Shen H., Kitt K., Liu T., Tao W., Du P. (2023). Spatiotemporal insight into early pregnancy governed by immune-featured stromal cells. Cell.

[194] Zang X., Zhang D., Wang W., Ding Y., Wang Y., Gu S., Shang Y., Gan J., Jiang L., Meng F. (2024). Cross-Species Insights into Trophoblast Invasion During Placentation Governed by Immune-Featured Trophoblast Cells. Adv. Sci..

[195] Sheikh B.N., Bondareva O., Guhathakurta S., Tsang T.H., Sikora K., Aizarani N., Sagar, Holz H., Grün D., Hein L. (2019). Systematic Identification of Cell-Cell Communication Networks in the Developing Brain. iScience.

[196] Lähnemann D., Köster J., Szczurek E., McCarthy D.J., Hicks S.C., Robinson M.D., Vallejos C.A., Campbell K.R., Beerenwinkel N., Mahfouz A. (2020). Eleven grand challenges in single-cell data science. Genome Biol..

[197] Rodriques S.G., Stickels R.R., Goeva A., Martin C.A., Murray E., Vanderburg C.R., Welch J., Chen L.M., Chen F., Macosko E.Z. (2019). Slide-seq: A scalable technology for measuring genome-wide expression at high spatial resolution. Science.

[198] Armingol E., Baghdassarian H.M., Lewis N.E. (2024). The diversification of methods for studying cell-cell interactions and communication. Nat. Rev. Genet..

